# Enhanced activity localization and microscale dosimetry in alpha-emitter radiopharmaceutical therapy using integrated autoradiography and histological imaging

**DOI:** 10.1038/s41598-025-09277-4

**Published:** 2025-08-14

**Authors:** Sandeep Sahota, Krystal A. Quirindongo, Joseph Piccolo, Anupriya Chhabra, Nouran Zaid, Simin Deveauxbray, Kenechukwu C. Charles-Obi, Brian Miller, Robert F. Hobbs, George Sgouros, Remco Bastiaannet

**Affiliations:** 1 Mallinckrodt Institute of Radiology, University of Washington in St. Louis School of Medicine, St. Louis, MO USA; 2 Russell H. Morgan Department of Radiology and Radiological Science, Johns Hopkins University School of Medicine, Baltimore, MD USA; 3 Department of Radiation Oncology, Department of Medical Imaging, College of Medicine, University of Arizona, Tucson, AZ USA

**Keywords:** Cancer therapy, Radiotherapy, Targeted therapies, Cancer imaging, Preclinical research, Translational research

## Abstract

Alpha-emitter radiopharmaceutical therapy delivers highly localized radiation, offering potent therapeutic effects. However, microscale heterogeneity remains poorly characterized in vivo and may affect efficacy. This underscores the critical need for sub-organ dosimetry to better understand αRPT radiobiology and guide treatment optimization. While autoradiography enables high-resolution activity mapping, conventional approaches lack anatomical context for accurate dose mapping. To address this, we propose a comprehensive workflow integrating quantitative autoradiography with histological imaging. Tissues from αRPT-treated mice bearing HER2 + breast tumors were snap-frozen, sectioned, and imaged using autoradiography. The same sections were histologically stained and used for precise autoradiography-histology integration. These anatomical contexts were then used to accurately stack multiple sections in a 3D volume, and were used for subsequent microscale dosimetry. Both tumor and kidney tissues were analyzed. Snap-freezing in isopentane preserved tissue morphology optimally. Our method enabled precise activity localization, revealing significant accumulation in the kidney cortex region close to glomeruli. Anatomical context improved 3D reconstructions needed for accurate dose estimations in tumor tissue. This methodology enhances αRPT dosimetry by precise spatial mapping of autoradiography unto the underlying tissue morphology. These advancements provide crucial insights into αRPT spatial radiobiology at the near-cellular level and will aid in optimizing radiopharmaceutical design and treatment planning.

## Introduction

The overall survival of patients with early-stage cancer, including breast cancer, has significantly improved over recent decades. However, for patients with distant metastases, prognosis remains poor despite advancements in treatment strategies^1,2^. Alpha-emitter radiopharmaceutical therapy (αRPT) has demonstrated remarkable efficacy in advanced-stage cancers, often leading to complete treatment responses when other therapeutic options have failed^3–5^. The high energy of emitted alpha particles is deposited within a very limited range of less than 80 μm, or less than a dozen cell diameters. This leads to densely clustered double-strand DNA breaks in irradiated cells while sparing the surrounding tissue. However, preclinical research has shown that the uptake of αRPT is generally highly non-uniform. This, combined with the short range of alpha particles, results in a significant fraction of the tumor receiving little radiation dose^6,7^. This shows that, while the overall efficacy of αRPT is evident, the underlying radiobiological mechanisms driving tumor response remain poorly understood. To address this, it is critical to perform dosimetry at the appropriate, near-cellular scale to accurately assess spatial radiobiological effects^8–12^.

Autoradiography is a widely used technique for imaging radiopharmaceutical distribution in tissue sections with high spatial resolution. However, conventional autoradiography methods often lack anatomical and histopathological context, making it difficult to precisely localize activity within specific sub-tissues. To overcome this limitation, some studies have used adjacent serial sections, with one section dedicated to autoradiography and another to histological staining^13–16^. However, this approach is hampered by sectioning artifacts such as tearing, folding, and warping, which make a precise fusion of autoradiography and histology images challenging. As a result, activity maps and histological images are often analyzed separately, introducing ambiguity in correlating radiopharmaceutical distribution with underlying tissue structures^13,15,16^.

Additionally, the short range of alpha particles in biological tissue presents a unique challenge for dosimetry. The thickness of standard tissue sections  (5–20 μm) is much smaller than the penetration range of alpha particles. As such, dosimetry must incorporate radiation penetrating from adjacent areas and cannot be performed based on a single 2D sections^16,17^. Therefore, accurate dosimetry requires three-dimensional (3D) reconstruction of activity distributions, which is complicated by the same sectioning artifacts that hinder autoradiography-histology alignment. Currently, no robust methodology exists to fully mitigate these artifacts and achieve precise 3D activity mapping. Integrating autoradiography with anatomical imaging could improve spatial localization and provide better insights into αRPT dosimetry at the microscale.

In this manuscript, we introduce a methodology that enables precise activity localization and microscale dosimetry by integrating autoradiography and histological imaging. By using the same tissue sections for both modalities, we ensure a direct 1:1 correspondence between radiopharmaceutical distribution and underlying tissue structures. Furthermore, our approach improves the spatial alignment by using multiple sections, reducing the effect of sectioning artifacts and allowing for more accurate 3D activity reconstruction. These advancements provide a powerful framework for studying αRPT response at a near-cellular level and optimizing treatment strategies.

## Methods

To achieve high-resolution activity localization and dosimetry, we developed a set of optimized techniques for tissue cryopreservation, sectioning, and imaging. Our approach ensures precise spatial matching between autoradiography and anatomical imaging, facilitating microscale dosimetry. We have specifically optimized tissue preparation, autoradiographic imaging and quantitative analysis. We have included some examples of applications of these techniques that are relevant to αRPT radiobiological research.

### Tissue cryopreservation & sectioning

Frozen sections are most often used for autoradiography, as the processing time required to make these sections is typically much shorter than the classical formalin fixed paraffin embedded (FFPE) tissue processing (~ 30 min vs. ~16 h)^18^ which helps with preserving the sample’s radioactivity. Additionally, the FFPE-method requires the sample to be permeated with formalin and paraffin, both of which could alter the original biodistribution through diffusion and displacement. Conversely, cryosectioning requires freezing of the tissue, which, if not properly controlled, can cause extensive freezing damage, presumably caused by expanding ice crystals ripping the tissue. This results in poor preservation of the tissue morphology^19–21^.

We therefore assessed the preservation of tissue morphology in kidneys using different methods for freezing fresh tissue which are often used in the literature, specifically: (i) in a -20 °C freezer or the cryostat’s Peltier element, (ii) using dry ice pellets, (iii) submerging in liquid nitrogen directly, and (iv) submerging in freezing isopentane, cooled in liquid nitrogen. No cryopreserving treatments (e.g. fixation, sucrose) were applied in any of the samples, as to not interfere with the isotope’s biodistribution. For this comparison, the tissues were allowed to equilibrate inside the cryostat for at least 2 h before sectioning started, to prevent temperature differences to confound our findings.

All tissues were sectioned at a thickness of 14 microns, adhered to positively charged microscope slides and used directly for autoradiography and/or subsequent imaging of the tissue morphology.

### Quantitative autoradiography

Autoradiographic images were acquired on the iQID MEGA camera system^15^ by placing the microscope slides with radioactive sections directly in contact with the manufacturer-recommended scintillator. The sections were then covered by a weight, ensuring even contact between the tissue samples and the scintillator.

The iQID system records individual detection events which were saved in list mode files. These files were processed using our in-house developed software, which implements corrections for: camera sensitivity, image warping, and can merge data in case of dropped frames or multiple acquisitions. The software outputs quantitative images of activity at any reference time in units of Becquerel, which were used for microscale dosimetry.

### Quantitative image generation from list mode data

The binary list mode files contain, amongst other data, the estimated centroid locations of each event, as well as a time stamp for each event. Images were created by binning events to a pixel grid, analogous to creating a 2D histogram. Specifically, events were binned into 26.5 μm × 26.5 μm square pixels to produce high-resolution activity maps. The time stamps were used to determine the total acquisition times and allowed us to splice together data from multiple recordings in case of unexpected acquisition issues, or for acquisitions longer than 60 h, which is the default maximum on the iQID device. Using the simple decay equation, and the camera sensitivity factor,$\:f$, we can derive the relation between number of decays and measured counts between time point 1 and 2:

$$\:Counts\left({t}_{1\to\:2}\right)={f\cdot\:N}_{0}\left({e}^{-\lambda\:{t}_{1}}-{e}^{-\lambda\:{t}_{2}}\right),$$

where $\:Counts\left({t}_{1\to\:2}\right)$ is the number of detected counts between time point 1 and 2, N_0_ is the number of radioactive atoms at reference time 0, $\:\lambda\:$ is the decay rate constant of the isotope in question. Now, if there are N recordings at different timepoints of the same sample, we can model combining these data, by simply summing the equation above for each acquisition interval i:

$$\:Count{s}_{total\:}=\:\sum\:_{i=0}^{N}Counts\left({t}_{i,1\to\:i,2\:}\right)=f\cdot\:{N}_{0}\sum\:_{i=0}^{N}\left({e}^{-\lambda\:{t}_{i,1}}-{e}^{-\lambda\:{t}_{i,2}}\right)$$

In this case, $\:Count{s}_{total}$ indicates the total acquired counts for a pixel or region of interest. This can be converted to activity at reference time 0 by using $\:A=\lambda\:N$. Rearranging results in:

$$\:{A}_{0}=\frac{\lambda\:}{f}\cdot\:\frac{Count{s}_{total\:}}{\sum\:_{i=0}^{N}\left({e}^{-\lambda\:{t}_{i,1}}-{e}^{-\lambda\:{t}_{i,2}}\right)}$$

This equation allowed us to determine the activity, A_0_, at any reference time, even when recordings were split in multiple sessions, and when using long acquisitions compared to the isotope’s half-life, without having to assuming constant (average) activity during the acquisition (i.e. total counts divided by acquisition time). For example, in the case of Ac-225, assuming constant activity over a 60-hour acquisition introduces only a ~ 0.25% error, but this increases to ~ 2% for a 1-week acquisition and nearly 8% for a 2-week acquisition—underscoring the importance of proper decay correction for longer (compared to the isotope half-life) measurements.

### Calibration

The camera was calibrated by imaging a series of dots of known activity. These dots were created by making a serial dilution of a calibrated activity concentration of Ac-225. A 2 µl drop of each dilution was put on a microscope slide which was placed on a heat block. After a couple of minutes, each dot was completely dried and adhered to the slide. The number of detected events was determined by making wide delineations around each spot on the acquired image and summing the total number of detected events.

The camera sensitivity factor, $\:f$, can then be found using $\:f=\frac{{n}_{measured}}{{n}_{primar{y}_{decays}}}$, where n_measured_ is the number of detected events, and $\:{n}_{primar{y}_{decays}}$ is the total number of isotope primary decays during the time of imaging. Consequently, this number may be larger than 1 for isotopes that emit multiple alpha particles per total decay of the parent isotope (e.g. the Ac-225 decay chain emits 4).

The detector efficiency is defined as the fraction of incident particles that are detected. To find this, we used that Ac-225 emits 4 alpha particles per primary decay, and that the activity was covered by the scintillator from one side, resulting in only 50% of particles ever hitting the detector. To get the detector efficiency, we therefore divided the sensitivity factor by 2 ($\:0.5\times\:4$ alphas).

### Image warping correction

To ensure accurate spatial alignment between autoradiography and microscopy images, we implemented a structured, multi-phantom warping correction procedure targeting distinct classes of geometric distortion in the iQID imaging system. First, we used a circles phantom provided by the vendor to determine and correct global isotropic scaling errors. The regular spacing of the circular features allowed us to calibrate the pixel spacing of the iQID detector relative to known physical dimensions. Second, a squares phantom, composed of equidistant rectangular elements, was used to assess and correct larger distortions across the field of view. Third, a dot pattern phantom was employed to identify and correct local warping. In each case, the idealized version of the phantom was digitally generated and used for non-rigid registration to the measured phantom image. This was implemented in SimpleElastix^22^ using a rigid transformation (2000 iterations), followed by a non-rigid b-spline transformation, two objective functions (normalized correlation, weight 1 and bending energy penalty, weight 0.5) and 5000 iterations. Each transformation was visually verified and found to be acceptable. This compound transformation functions as the warping correction from a distorted raw iQID image to a corrected image.

All warping corrections were applied to autoradiography images prior to any further registration steps. The resulting transformation fields were used to correct each quantitative autoradiography image. To preserve intensity accuracy, the transformed images were corrected using the determinant of the Jacobian matrix of the total deformation field to account for local area density changes. Following this correction, no further scaling adjustments were permitted during microscopy-to-autoradiography coregistration, ensuring that the autoradiography pixel grid corresponded to physically calibrated spatial dimensions.

### Integrating autoradiography and morphological imaging

Integration between autoradiography and morphological imaging was achieved by imaging the exact same sections twice: once on the autoradiography device, then after staining, again for morphological imaging. Because the two images are of the same sections, in principle, there now exist a perfect 1-to-1 mapping between the two images. However, perfectly lining up the two imaging modalities is challenging, because: (i) the activity biodistribution does not necessarily follow tissue morphology (e.g. active tissue has surrounding connective tissue), so the spatial correspondence might not be immediately obvious, (ii) tissue sections, especially smaller tumors, may have symmetries, leading to difficulties resolving rotations, and (iii) staining the slide that has been on the autoradiography device for potentially days to weeks is non-trivial.

To mitigate these issues, we placed 3 sections on each microscope slide that were imaged together as a group on both modalities. As such, the relative locations of the sections were fixed, addressing the issues mentioned above by increasing the rigid registration’s ability to resolve small errors, particularly rotations. We used a two-step registration procedure implemented in SimpleElastix. First, manual landmark initialization was performed using three reference points and solved using a direct least-squares fitting algorithm^23^. This was followed by a rigid Euler transformation optimized over 4000 iterations. All other registration parameters were left at default. Registration quality was assessed visually and confirmed by the plateauing of the objective function (mutual information), indicating convergence.

Morphological imaging after autoradiography involved fixation with freshly prepared 4% paraformaldehyde (PFA) followed by staining with Phalloidin-Alexa Fluor 633 and Hoechst (P&H). Phalloidin staining visualizes filamentous actin (F-actin), a critical component of the cytoskeleton, allowing for detailed cellular structure visualization. Hoechst staining highlights the nuclear architecture. Together, these stains provide a clear assessment of cellular boundaries and tissue organization, analogous to the Hematoxylin & Eosin (H&E) stain commonly used in pathology. The slides were imaged on a Zeiss AxioScan 7 slide scanner, and the images were false-colored to closely resemble standard H&E stains, following a previously published method^24^.

**Fig. 1:**
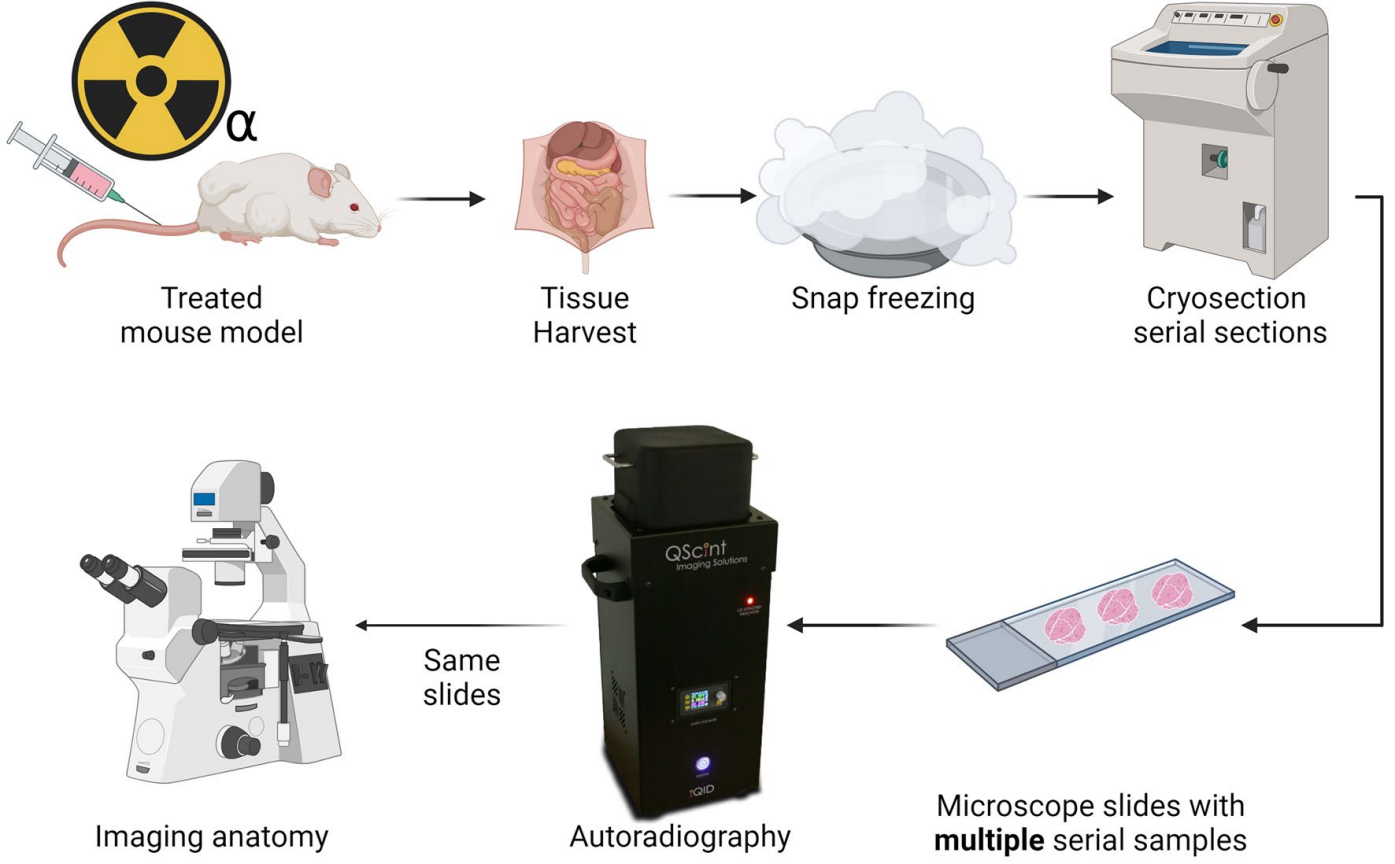
Workflow of integration of autoradiography with underlying anatomy. After harvesting, the tissues were snap-frozen, and at least 3 sections were adhered to a microscope slide and imaged on the autoradiography device. The same sections were then stained for morphology and subsequently imaged fluorescently (P&H). By combining multiple sections on a microscope slide, the locations of each section, relative to each other, were fixed between autoradiography and morphological imaging. Consequently, there exists a perfect 1:1 (rigid) mapping between the two imaging modalities, which aides creating a near-perfect overlay between the two by increasing the robustness of the coregistration, particularly against rotation and translation imperfections.

An overview of the data acquisition workflow is given in Fig. 1.

### Mouse treatment protocol

The tissue sections utilized in this study were obtained from a broader experiment involving the syngeneic immunocompetent Neu-N mouse line, which was subcutaneously implanted with the HER2-expressing NT2.5 cell line^25–27^. Ten days post-implantation, when the tumors became palpable, the mice were administered an anti-HER2 antibody labeled with Ac-225, with a total administered activity of 14.8 kBq. The radiolabeling protocol employed is detailed elsewhere^28^. Tumors were harvested at predefined time points, including one at 3 days post-injection (pi). For biodistribution analysis, blood, liver, and kidney samples were collected in triplicate at 22 h and 27 days pi, weighed, and measured using a calibrated gamma counter to determine percent injected activity per gram of tissue (%IA/g). To further characterize renal uptake patterns, two additional non-tumor-bearing mice were injected with 74 kBq of free Ac-225 and their kidneys harvested at 1 and 6 h pi. No control group was included in this study.

### Ethical approval

All procedures were approved by the Washington University in St. Louis Institutional Animal Care and Use Committee (animal welfare assurance number D16-00245). All experiments were performed in accordance with all relevant guidelines and regulations. The authors have adhered to the ARRIVE guidelines.

### Microscale Spatial analysis of kidney uptake

A distinct pattern of activity uptake in the kidney cortex after RPT has previously been observed by multiple authors^29,30^. As the kidney’s function is spatially organized from cortex to medulla, it is believed that this pattern is related to some cortical filtration function. However, the exact mechanisms are likely to be specific to the exact isotope and targeting agent^30^. As such, this phenomenon is not well-understood^31^.

To that end, we analyzed the spatial uptake patterns in kidney samples of treated mice at 22 h and 27 days pi. The samples were processed, stained, and imaged as described above. Using SimpleITK^32^ the phalloidin scans of these kidney sections were segmented into 3 compartments: (i) entire tissue, (ii) blood vessels, and (iii) glomeruli by using simple thresholding methods (Otsu^33^), and binary mask operations: connected component analysis, followed by using mask shape statistics such as size, perimeter, and bounding box size to distinguish between the compartments. We applied a Euclidian distance transform to these binary images, which allowed us to plot activity versus distance from each compartment. We combined data from 3 sections of 3 individual kidneys (9 total) per time point and compared the spatial uptake patterns between the two timepoints. To test for statistical significance, 95%-confidence intervals were calculated. Whenever the average of one condition exceeded these intervals, this was considered a significant difference (*P* < 0.05).

### Near cellular-level dosimetry

Dosimetry, distinct from activity localization, requires three-dimensional (3D) information because radiation from adjacent sections contributes to the absorbed dose in a given tissue region. This necessitates reconstruction of a 3D activity volume. Traditionally, such reconstruction is achieved by stacking activity maps from serial sections,^16^ though this process is prone to misalignment due to sectioning artifacts. In our approach, we leveraged co-registered anatomical (P&H) images corresponding to each activity map to guide alignment. These higher-fidelity images contain clearly defined tissue boundaries, cavities, blood vessels, and other landmarks that support more robust non-rigid registration. Visual assessment of the anatomical overlays further supported superior alignment compared to activity maps alone.

To reconstruct 3D activity volumes for dosimetry, we co-registered three adjacent sections per slide. Initial alignment was achieved using a three-point landmark-based initialization, as described above. We implemented and compared two stacking strategies: one, previously described, based on activity maps alone, and our proposed method using anatomical images for guidance. In the activity-only approach, registration consisted of a rigid Euler transformation (2500 iterations) followed by a similarity transformation (4000 iterations) using a single resolution level. In the anatomy-guided approach, an additional non-rigid B-spline transformation was applied, using a multiresolution grid spacing schedule of 150×, 75×, 30×, and 15× the microscope pixel spacing across four pyramid levels. Optimization was driven by a weighted combination of mutual information (weight = 1.0) and bending energy penalty (weight = 2.5), with a maximum of 2000 iterations. Convergence was informally verified by monitoring the plateauing of the objective function, and all results were visually inspected for anatomical plausibility and absence of over-deformation and folding.

To quantitatively evaluate the effectiveness of these stacking strategies, we compared their performance using the Dice similarity coefficient (DSC) as a metric of spatial overlap for a total of 14 tumor sets. For both approaches, the resulting transformation fields were applied to the anatomical (P&H) images, which were segmented using thresholding and connected component analysis. DSCs were then computed between anatomical masks of one adjacent section pair after registration. In the anatomy-guided approach, non-rigid registration was performed directly on the anatomical images. In the activity-only approach, registration was performed on the activity maps, and the resulting transformations were applied to the anatomical images for evaluation. A paired t-test was used to determine whether the anatomy-guided registration provided a statistically significant improvement in spatial alignment.

Dose-rate maps were generated by convolving a dose-voxel kernel (DVK) with the quantitative activity maps, as previously described^16^. The DVK was generated in GEANT4 using a grid of voxel detectors with dimensions matching the iQID pixel spacing and tissue section thickness. A total of 1 × 10⁶ primary decays were simulated from locations randomly distributed within the central voxel. Energy deposition in surrounding voxels was averaged to construct the kernel. The DVK assumed water-equivalent tissue; due to the short particle ranges involved, minor density variations were not modeled, as they are unlikely to significantly influence local dose estimates at the microscale.

## Results

### Tissue cryopreservation

**Fig. 2:**
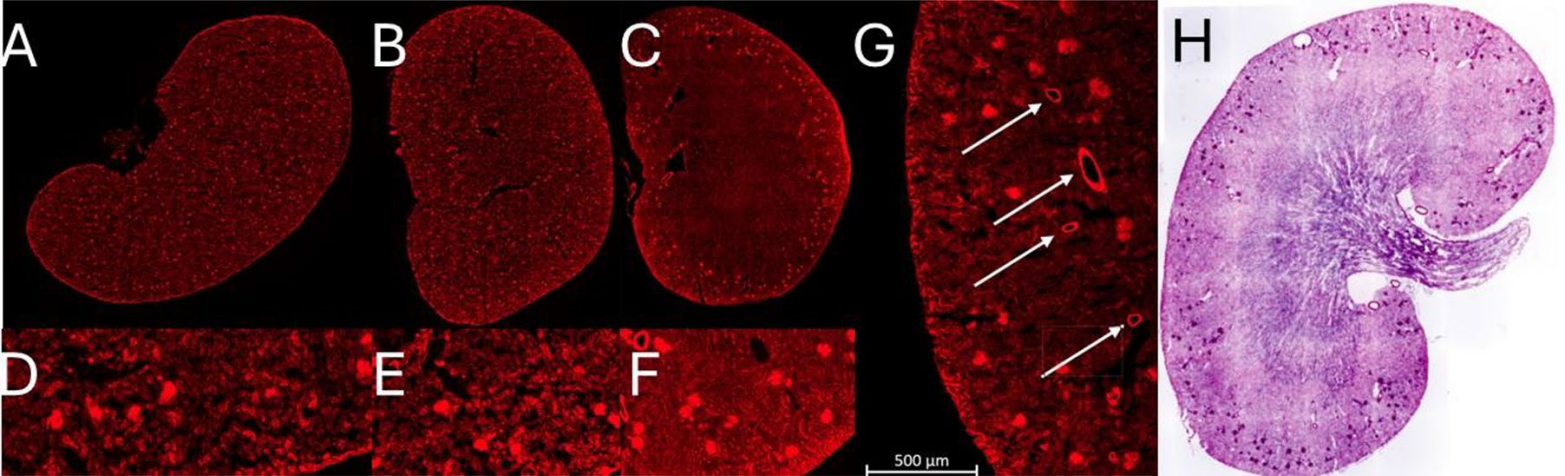
The extent of freezing damage on kidney tissue stained with phalloidin when cryopreserving by: (A) placing embedded tissue on dry ice, resulting in extensive damage causing ripped tissue, with no discernable tubular structure left (detail in D); (B) using − 20 freezer or Peltier element in cryostat, further exacerbating the observed freezing damage (detail in E); (C) Snap-freezing in isopentane chilled in liquid nitrogen, preserving the morphology of glomeruli and tubules (detail in F). (G) Major vasculature is readily discernable with phalloidin stain of snap-frozen kidney tissue. (H) P&H stain false-colored to look like a normal histological H&E stain.

The impact of freezing damage caused by several commonly used freezing methods is shown in Fig. 2. Snap-freezing by directly submerging the tissue block into liquid nitrogen often resulted in deep cracks, preventing complete sections to be made reliably (not shown). Snap-freezing in isopentane reliably preserved tissue morphology, which allows for the identification of tissue sub-structures on microscopy images. We routinely observed preservation of the epithelial lining of vasculature using this method only (Fig. 2G).

### Autoradiography: image warping correction

**Fig. 3:**
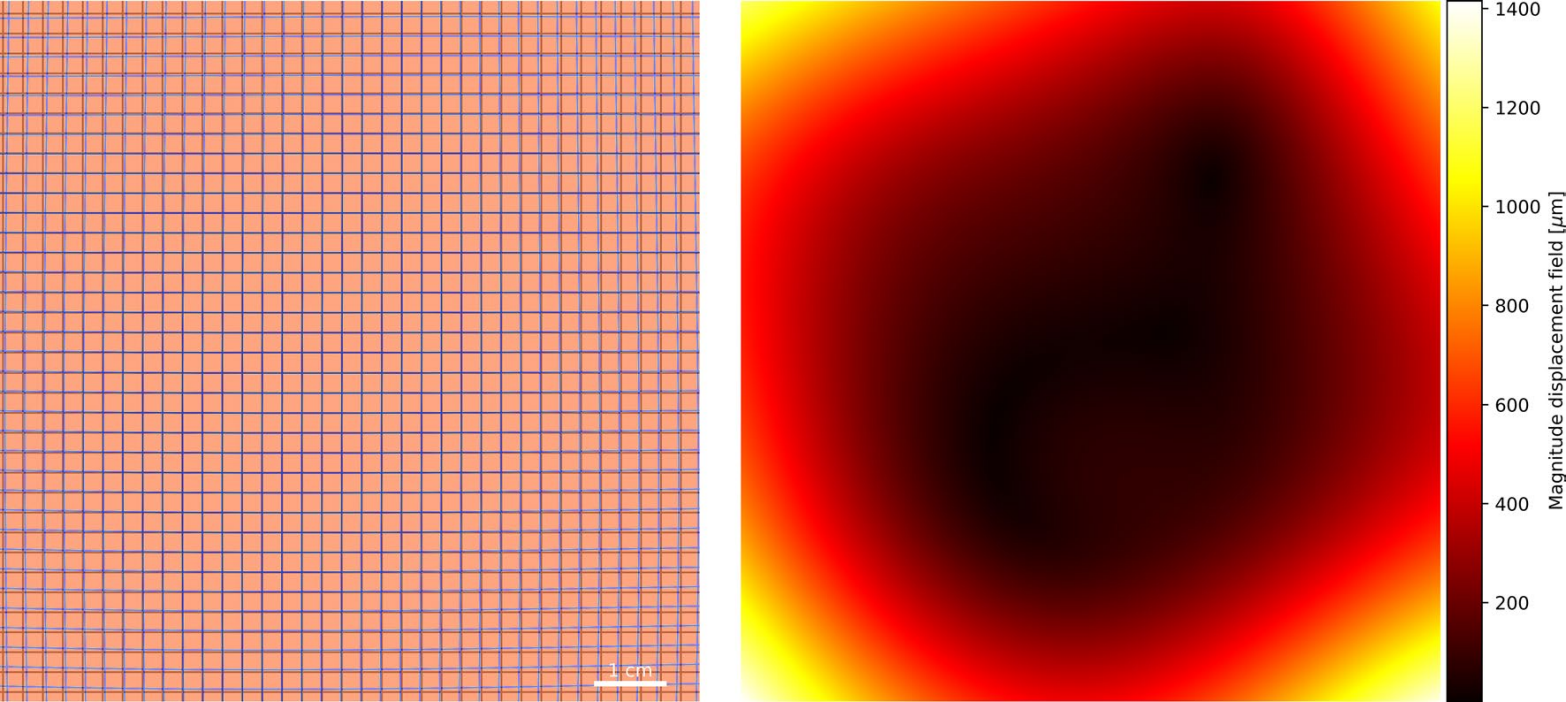
The magnitude of the displacement between measured and perfect pattern phantoms is indicative of the extend of image deformation on the iQID MEGA. The largest displacements are found in the corners of the image.

The extent of image deformation in the raw iQID images is shown in Fig. 3. Global isotropic pixel scaling mismatches were revealed by the circles phantom and quantified at 1.6%. In addition, spatial distortion was evident in the corners of the field of view, with displacements exceeding 1 mm.

### Integrating autoradiography and morphological imaging

**Fig. 4:**
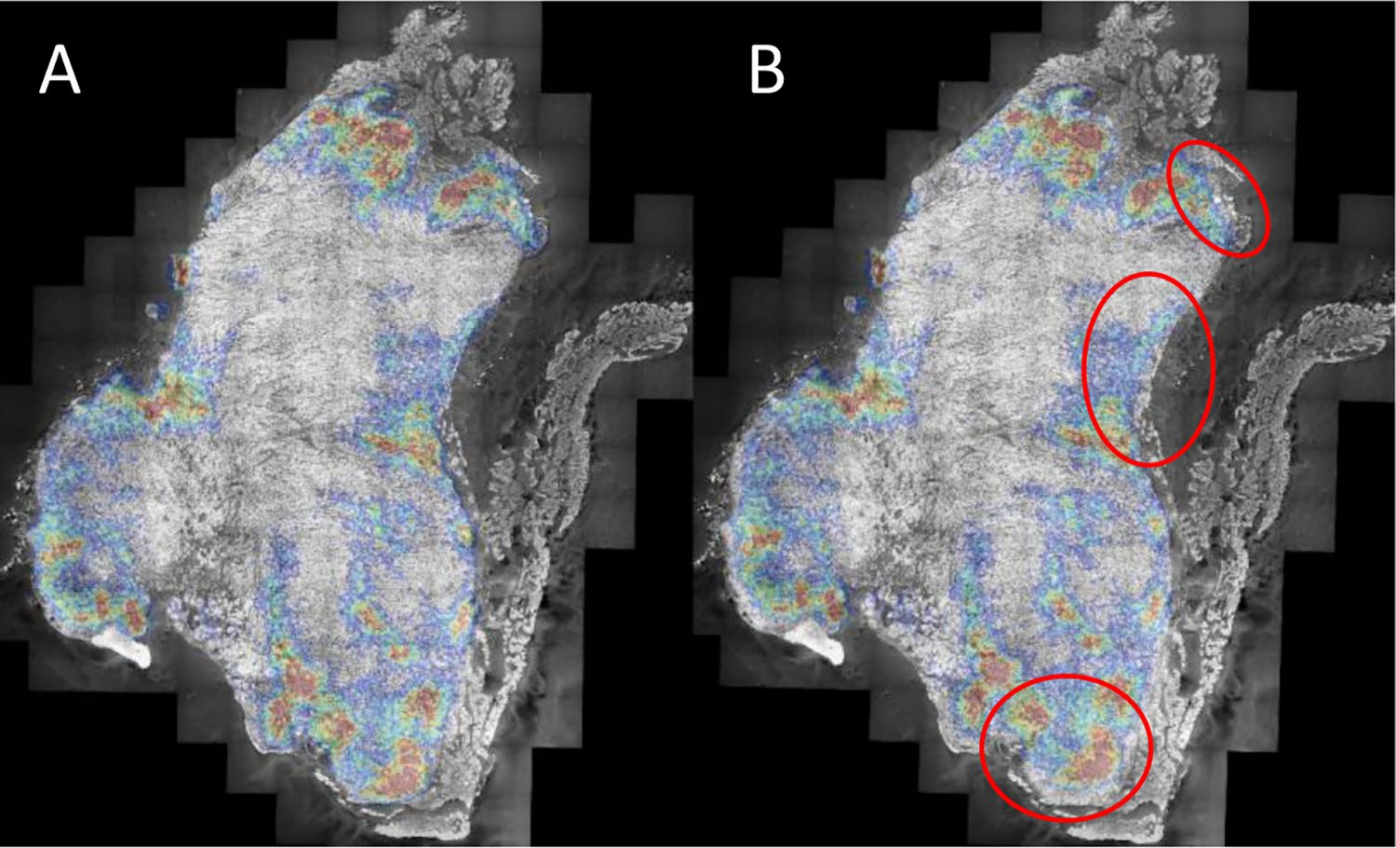
Coregistration quality when using 3 or 1 sections per slide for a coregistration. Images showing activity (color) superimposed on gray-scale (P&H). Small registration errors are resolved when using 3 sections (A), but not when using a single section (B) for registration. Significant mismatches shown along the outside tissue border in (B) (circles). This is a typical example in which the activity does not perfectly follow the tissue outline due to additional non-tumor (e.g. connective) in the section. Tumor recording from 3 days pi. P&H image is displayed in gray scale to increase clarity.

A typical example of the improved robustness achieved by registering anatomical to autoradiography images using three, rather than a single, tissue section is shown in Fig. 4. When only one section was used, registration frequently resulted in rotational mismatches and less stable alignment, particularly in tissues with ambiguous or symmetric morphology. Including three sections per slide introduced fixed spatial relationships that provided additional geometric constraints, which seemed to improve the accuracy of rigid registration.

### Microscale spatial analysis of kidney uptake

**Fig. 5:**
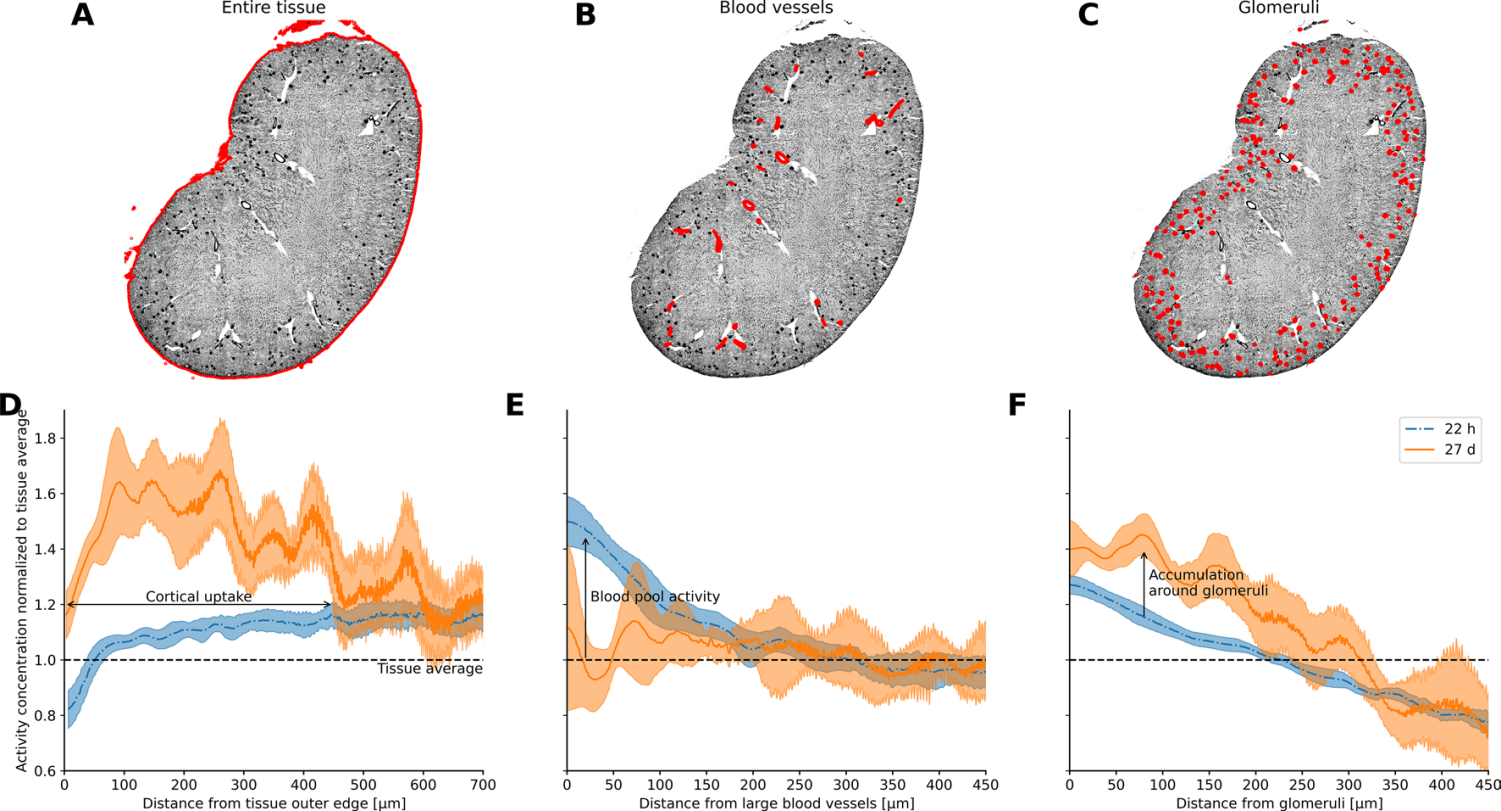
Spatial analysis kidneys at 22 h and 27 days post-injection (pi). For each time point, 3 sections from 3 individual kidneys were included. Panels A-C show example segmentations of the tissues of interest on the phalloidin images. D) Uptake as a function of distance from outer tissue edge, relative to average activity concentration in the tissue section. At 27 days pi, a statistically significant accumulation in the cortex, but not the deeper medulla, is readily discernible. E) The blood pool activity concentration was significantly above tissue average at 22 h pi, but not at 27 days pi. F) The activity concentration around the glomeruli was significantly elevated at both 22 h and 27 days pi relative to the tissue average. At the later time point, the accumulation increased further, peaked at 75 micrometers, and was significantly elevated between ~ 5–200 micrometer distance from the glomeruli, relative to the earlier timepoint, indicating a mechanism of accumulation proximal (but not inside) the glomeruli. This effect cannot be explained by general cortical increase, or spill-over from the blood pool activity. Shaded areas indicate 95%-confidence interval.

After radiolabeling, the radiochemical purity was measured to be 98.4%. A clear statistically significant (*p* < 0.05) increased uptake in the cortex of mice treated with 14.8 kBq of our agent is shown in Fig. 5A/D. Figure 5 panels B/E show that the relative contribution of the blood pool (cortical and otherwise) to the average activity went down with time and was insignificant at 27 days pi. Figure 5 panels C/F demonstrate that the glomeruli themselves did not increase their relative uptake much over time, but that there was a significant (*p* < 0.05) increase slightly away from these structures. This shows that not the blood pool, but some structure close to the glomeruli -and not the glomeruli themselves- are involved in the observed cortical uptake patterns.

**Fig. 6:**
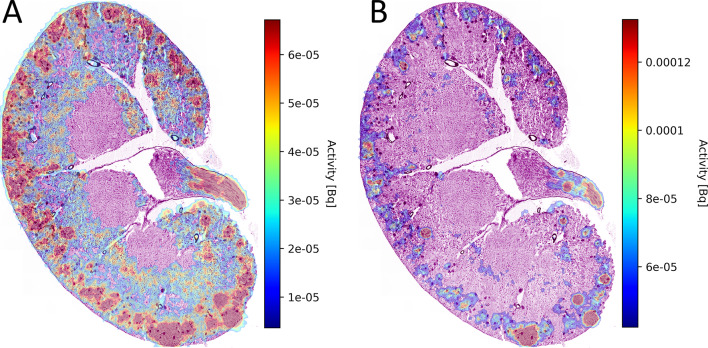
Kidneys of a mouse injected with 74 kBq unbound Ac-225. Harvested at 6 h pi. (A) Overlay showing strong cortical uptake. (B) Same data, showing only areas of high activity. A pattern of activity accumulation mostly close to, but not perfectly overlapping with glomeruli (dark round spots) is visible. Activity is expressed in activity per pixel.

As antibodies are typically not filtered by the glomerulus^31^ and given that the time between animal sacrifice and autoradiography exceeded two hours (eliminating contributions from Ac-225 progeny due to short-range decay displacement), we hypothesized that the cortical activity may have resulted from the accumulation of unbound Ac-225 or radiometal-containing catabolites. To visualize the potential spatial pattern of such uptake, we injected a non-tumor-bearing mouse with 74 kBq of unbound Ac-225—fivefold higher than the therapeutic dose—and imaged the kidneys at 6 h post-injection. The resulting distribution, shown in Fig. 6, illustrates a comparable cortical uptake pattern.

To complement the spatial analysis, we performed biodistribution measurements in these mice at 22 h and 27 days post-injection. At 22 h, measured activities were 15.7%IA/g in blood, 28.6%IA/g in liver, and 9.7%IA/g in kidney. By 27 days, blood activity had declined to 0.2%IA/g, while kidney and liver retained 1.4%IA/g and 12.2%IA/g, respectively.

### Near cellular-level dosimetry

#### Device calibration

**Fig. 7:**
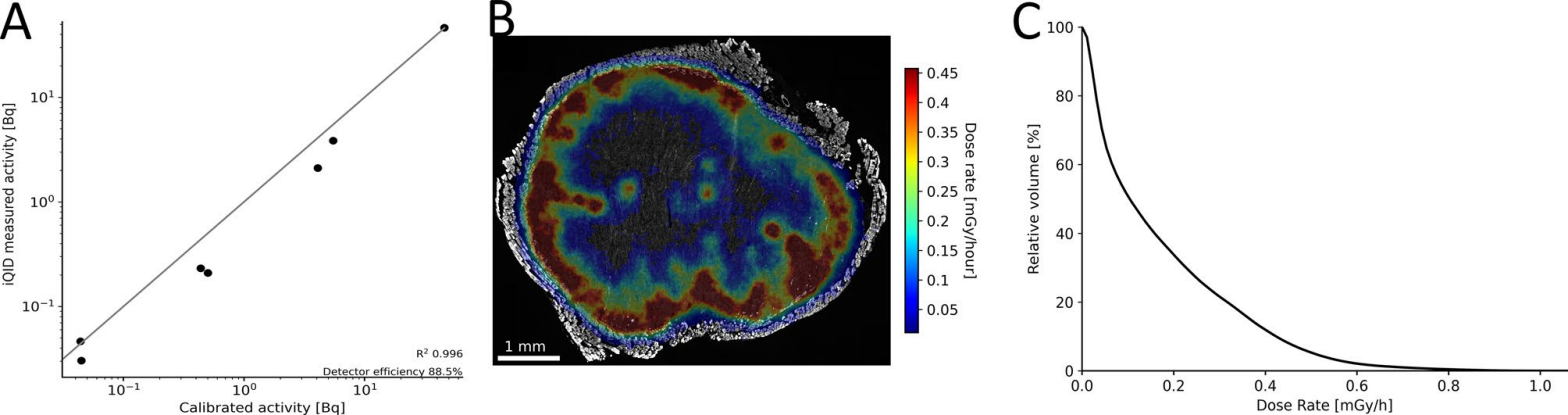
Aspects of near-cellular dosimetry. (A) iQID absolute activity quantification after calibration with activity in multiple orders of magnitude, showing a good correlation (high R^2^) between calibrated activity and measured activity. Line shown is identity (y = x). (B) Example of dosimetry for a tumor harvested at 3 days pi. Dosimetry based on anatomy-based volume reconstruction of 3 sections, spaced 28 μm apart. Dose rate map perfectly followed tissue outline, indicating a good spatial alignment. Dose rate was highly non-uniform, as is typical for (antibody-based) αRPT. (C) The corresponding cumulative dose-volume histogram, further illustrating the dose rate non-uniformity.

Serial dilutions were made of an aliquot of actinium nitrate solution with a calibrated activity concentration. Drops of these dilutions were dried onto a microscope slide and measured on the iQID for a total of 82 h. The camera sensitivity factor, $\:f$, was found to be 1.77 through linear regression of the measured activity onto the calibrated activity (Fig. 7A). The estimated detector efficiency for our device and settings was 88.5%.

#### 3D volume reconstruction

Proper dosimetry requires that a 3D volume of activity surrounding the target volume (i.e. section) is reconstructed. To quantitatively assess the alignment quality of our 3D reconstruction approach, we compared our proposed anatomy-guided and previously suggested activity-only stacking using the Dice similarity coefficient (DSC) on anatomical masks. The DSC was calculated between a pair of adjacent anatomical sections following registration. The anatomy-guided method, yielded an 8.6% higher Dice coefficient compared to the activity-only method. This improvement was statistically significant (*p* = 2.379 × 10⁻³, paired t-test), supporting the conclusion that anatomical image guidance improves spatial alignment between sections.

An example of a fully calibrated (Fig. 7A), and executed microscale dosimetry is given in Fig. 7B. Even though the diameter of this tumor was about 0.5 cm, the very non-uniform dose rate is readily discernible in panel B. The corresponding cumulative dose-volume histogram is given in Fig. 7C, showing that 50% of the tissue received less than 10% and 25% of the tissue less than 3% of the maximum dose rate.

## Discussion

In this study, we developed and validated a comprehensive methodology to improve autoradiography-based activity localization and microscale dosimetry for αRPT. Our approach successfully addresses key limitations in traditional tissue sectioning, imaging, and registration techniques, thereby enabling more accurate spatial analysis of radiopharmaceutical uptake and its dosimetric implications.

Autoradiography techniques to study microscale dosimetry in αRPT have recently gained significant attention^34^ especially with a focus on Radium-223 chloride (Xofigo) localization in the trabecular bone^35–38^ Astatine-211 in prostate cancer^39^ ovarian cancer^40^ and several deconvolution techniques (resolution recovery)^41,42^. However, there is currently no literature on a comprehensive and robust set of methods dealing with tissue preparation, sectioning, fusion of multimodal images, and microscale dosimetry. The current manuscript deals with all these aspects.

We demonstrated that freezing techniques significantly impacted tissue morphology preservation. Our findings indicate that snap-freezing in isopentane cooled with liquid nitrogen was the most effective method we tested for maintaining structural integrity while minimizing freezing artifacts. Other snap-freezing methods (e.g. isopentane chilled in a dry ice/ethanol slush) may yield similar results. In contrast, methods relying on putting a sample on dry ice or in a − 20 °C freezer resulted in substantial tissue damage, leading to unreliable histological assessments. While this distinction may be less critical for course organ-level activity localization, it is crucial for high-resolution dosimetry in smaller substructures, where preserving anatomical detail is essential. We acknowledge that our work is primarily focused on soft tissue tumors and this protocol would probably need to be amended for bone tumors, as this hard tissue requires a different set of tissue preparation and sectioning methods.

Our proposed set of methods was successful in directly integrating autoradiography with histological imaging by staining and imaging the same tissue sections. This approach eliminates the common problem of sectioning artifacts that hinder accurate coregistration when using adjacent sections for different imaging modalities. By placing at least three sections per microscope slide, we ensured fixed spatial relationships between sections, thereby allowing for presumably more robust coregistrations.

The image warping corrections applied to the iQID system were critical to the accuracy of activity localization. The observed deformation pattern is consistent with the remnants of barrel distortion, commonly seen in wide-angle optical systems^43,44^. Our measurements showed that, without correction, distortions—particularly at the image periphery—could lead to localization errors exceeding 1 mm, which is unacceptable for near-cellular dosimetry. Correcting for this warping is therefore essential when generating precise overlay images. By using vendor-provided calibration phantoms, we achieved high-fidelity spatial corrections, substantially improving the accuracy of our methods.

To demonstrate a potential application of our method, we performed a spatial analysis of kidney uptake patterns following αRPT. While individual autoradiographic images of the kidneys were inherently noisy due to the low administered activity (14.8 kBq), our spatial analysis pipeline enabled us to extract statistically significant and biologically meaningful uptake trends by aggregating data across multiple sections. We observed a distinct and significant increase in activity near glomeruli over time, while blood pool activity—localized primarily to large vessels—decreased. This opposing trend supports the conclusion that the near-glomerular accumulation is not due to residual blood signal or spill-over but rather reflects a separate underlying retention mechanism. We did not perform dosimetry on these structures, as doing so would require reliable segmentation of proximal tubules across serial sections—a task that remains challenging with current automated methods and was beyond the scope of this study.

Although the segmentation of blood vessels was limited to larger vessels, this was by design: complete resolution of the renal microvasculature, including glomerular capillaries, was beyond the capabilities of our imaging approach and unnecessary for this analysis. Instead, segmenting large vessels served to reduce partial volume effects and clearly delineate the blood pool compartment. The spatial and temporal separation between compartments suggests that the retained activity near glomeruli likely represents uptake by adjacent structures, with the proximal tubules as a plausible candidate^45^. This interpretation is further supported by biodistribution data from the same cohort, which show that kidney uptake, initially lower than blood at 22 h (9.66% vs. 15.73% IA/g), becomes higher by 27 days post-injection (1.44% vs. 0.21% IA/g), indicating an accumulation or retention mechanism. Although the liver (and presumably bone) remained a more dominant site of retention, kidney activity was clearly non-negligible and persistent over time.

Autoradiography was performed at least two hours post-sacrifice, minimizing any contribution from translocated Ac-225 daughters. Additionally, while the injected radiopharmaceutical exhibited high radiochemical purity (98.4%) and has been shown to be stable in buffer at 37 °C^46^ it is well known that radiolabeled antibodies undergo in vivo catabolism via FcRn-mediated recycling and lysosomal degradation^47^. We therefore suspect that the renal accumulation results from physiological breakdown and renal handling of the construct rather than from passive instability in circulation alone. A distinct band of activity observed in the outer stripe of the outer medulla (Fig. 6A) is consistent with prior reports of localized radiation-induced damage in this region^48^. Together, these findings highlight the importance of considering in vivo catabolism and isotope clearance mechanisms when designing and interpreting αRPT toxicity studies. They also demonstrate the strength of our statistical approach in extracting biologically meaningful patterns from low-activity, high-noise autoradiographic data. In future work, it would be valuable to relate these microscale renal uptake patterns to whole-organ and whole-body distributions, particularly when interpreting off-target toxicity and comparing retention across organs such as the liver, kidney, bone, and bone marrow.

A key advancement in this study is the integration of autoradiography with anatomical imaging for 3D volume reconstruction, which is critical for accurate microscale dosimetry. By using co-registered anatomical (P&H) images to guide 3D stacking, we improved the robustness of section alignment compared to activity-based methods, which are susceptible to distortions from sectioning artifacts and sparse uptake patterns. Quantitative analysis confirmed that the anatomy-guided approach yielded an 8.6% higher Dice similarity coefficient relative to activity-based registration, with this difference reaching statistical significance (*p* = 2.379 × 10⁻³). Although dose-rate volume histograms (DVHs) are commonly used to summarize spatial dose distributions, they are not reliable indicators of registration accuracy in sparse datasets, where activity-based methods tend to overemphasize alignment of isolated hotspots. In contrast, anatomical images offer consistent structural landmarks across sections, supporting more accurate spatial correspondence and yielding a more reliable foundation for microscale dosimetry—particularly in small or morphologically complex tissues. Our strategy builds on earlier film-based reconstruction methods^49,50^ by enabling high-resolution multimodal image fusion with enhanced spatial fidelity.

Despite these efforts, it is important to recognize the inherent limitations in evaluating registration performance at the microscale. In the absence of a true ground truth, metrics such as the Dice similarity coefficient or landmark distance offer useful—but imperfect—proxies for alignment quality. Furthermore, such metrics are sensitive to tissue morphology, segmentation accuracy, and sectioning artifacts, all of which introduce uncertainty. While our anatomy-guided method outperformed activity-based registration by these measures, we acknowledge that absolute validation of spatial accuracy remains challenging. Future work may benefit from synthetic phantoms or fiducial markers to further benchmark registration algorithms in this context.

The methodological advances presented here have practical implications. Our dosimetric analysis revealed pronounced heterogeneity in absorbed dose distributions from antibody-mediated αRPT, even in small tumors (~ 0.5 cm in diameter). Large fractions of tissue received only a small percentage of the peak dose. Bulk-tissue dosimetry, which averages over these spatial variations, risks misestimating the therapeutic implications of these microscale heterogeneity^11^, further underscoring the critical need for high-resolution, near-cellular level dosimetry in evaluating α-particle therapies^49^.

## Conclusion

This study presents a practical and reproducible methodology for high-resolution activity localization and microscale dosimetry in ex vivo tissue sections of subjects treated with αRPT. Our approach provides a framework for investigating spatial radiobiology with improved accuracy, which is essential for optimizing radiopharmaceutical design and treatment planning.

Future work should focus on the biological mechanisms driving spatial uptake heterogeneity, which will be crucial for improving the therapeutic index of αRPT agents. By leveraging our proposed methodology, it is now possible to systematically study tissue penetration rates, radiation-induced damage, and cellular responses to α-emitter therapy at a near-cellular scale.

## Data Availability

Analysis code related to this manuscript can be found on Github: https://github.com/RemcoBastiaannet/iQIDParser.
